# The influence of host genetics on the microbiome

**DOI:** 10.12688/f1000research.20835.1

**Published:** 2020-02-05

**Authors:** Alexandra Tabrett, Matthew W. Horton

**Affiliations:** 1 Plant and Microbial Biology, University of Zurich, Zurich, CH-8008, Switzerland

**Keywords:** Host microbiome, plant microbiome, beta diversity, microbes, microbial communities, genetic influence, Microbiome traits, metabolism, plant, plant genetics

## Abstract

It is well understood that genetic differences among hosts contribute to variation in pathogen susceptibility and the ability to associate with symbionts. However, it remains unclear just how influential host genes are in shaping the overall microbiome. Studies of both animal and plant microbial communities indicate that host genes impact species richness and the abundances of individual taxa. Analyses of beta diversity (that is, overall similarity), on the other hand, often conclude that hosts play a minor role in shaping microbial communities. In this review, we discuss recent attempts to identify the factors that shape host microbial communities and whether our understanding of these communities is affected by the traits chosen to represent them.

## Introduction

Microbiome studies often focus on bacteria. However, microbial communities encompass all of the microorganisms in a particular environment. These can include yeasts, filamentous fungi, oomycetes, bacteria, archaea, algae, protists, viruses, nematodes, and even small arthropods. A microbiome, and the interactions within these communities, can directly or indirectly affect a host’s health, development, and physiology. In plants, for example, microbes influence important fitness and developmental traits ranging from disease resistance ^1^ to flowering time ^2^. In animals, the microbiome has been shown to influence nutrient uptake ^3^, abiotic stress tolerance ^4^, and even the development of the central nervous system ^5^.

Given the role of microbiota in host health and phenotypic variation, several studies have sought to understand how environmental factors, interactions among microbes, and host genetic differences shape these communities. Whereas some studies have concluded that genetic differences among hosts influence microbiota ^6– 13^, others have concluded that hosts play at most a minor role ^14– 16^. The discrepancy in results may arise from differences in perspective: is the proverbial glass (here, the glass contains the heritability of the microbiome) half empty or half full? Differences in study design and methodology also appear to sway results. Perhaps tellingly, studies of model organisms reared in environmentally controlled conditions regularly conclude that microbial communities are under some level of host control ^7, 11, 17, 18^; the relationship becomes less clear, however, in environmentally complex field settings ^13, 16^.

Here, we discuss recent research focused on understanding whether, and to what extent, host microbial communities are under the influence of host genes. Overall, the results from these studies often appear to depend on the approaches used to characterize microbial communities. Therefore, we provide an overview of the most commonly used microbial traits and the possible pitfalls of using each phenotype.

## Microbiome traits

Microbial communities are typically summarized by using one or more of four possible methods. Analyses of beta diversity, alpha diversity, or the abundances of individual taxa are usually conducted after the polymerase chain reaction (PCR) and sequencing of phylogenetically conserved regions, known as marker genes. The most commonly sequenced regions include sections of ribosomal RNA (rRNA) genes or the internal transcribed spacers (ITSs) found in eukaryotic (for example, fungi) DNA. The fourth strategy, characterizing microbial metabolism, is less common; this is likely due to the costs of characterizing microbial communities by using shotgun sequencing or metatranscriptomic approaches.

### Beta diversity

Beta-diversity metrics quantify the overall similarity among samples due to spatial differentiation or other mechanisms, and, given the multivariate nature of microbiome data, these measures have become widely used to understand the factors that influence microbial communities. Perhaps the best known index is the “semi-metric” developed by the ecologists Bray and Curtis ^19^. Alternatively, one can use the phylogenetic distance metric UniFrac, which measures the evolutionary divergence among microbial communities by using a phylogenetic tree constructed from a multiple sequence alignment ^20^. Jaccard, Kulczynski, Euclidean, Hellinger, and chi-squared–based distances are other commonly used measures ^21^.

Each of these indices has strengths and weaknesses. As an example, phylogenetic distance methods, such as UniFrac, require high-quality sequence alignments that are difficult to generate and curate during large-scale sequencing projects. Notably, high-quality alignments are especially difficult to generate using marker genes that contain sequence-length polymorphisms, such as the ITS regions found in eukaryotic (for example, fungal) DNA. Many (but not all ^22^) metrics are also sensitive to differences in the number of sequencing reads among samples ^23^, making it necessary to either normalize the data (for example, by expressing the abundances of data as a proportion or a log-ratio) or resample the data to a given read count by using the observed probability distribution within the sample. Identifying robust beta-diversity metrics, and the optimal preprocessing steps, is an area of ongoing research.

Primer mismatches and variation in the number of marker genes among species ^24^ pose additional problems (for all microbial analyses) and have been implicated in poor reproducibility among sequencing runs ^25, 26^. Yet another challenge is that zeros are common in species data; that is, many taxa are observed in only one or a few samples. The problem of sparse species data has been discussed in the community ecology literature for decades ^27^, but the zeros in microbiome data are particularly difficult to address because they may reflect the real absence of an organism in a sample (that is, a true zero) or they may be the consequence of undersampling and sequencing artifacts.

Some of the most widely used measures of beta diversity were developed by botanists ^19, 28^ investigating whether differences among plant communities across field sites could be attributed to different aspects of the environment. Unfortunately, it is unclear whether the species concept can be applied to prokaryotes, which regularly acquire DNA from the environment through horizontal gene transfer (HGT) mechanisms that blur species boundaries ^29^. The frequency of HGT differs across spatial scales ^30^, but it is common enough among prokaryotes that pairwise genome-wide DNA similarity and 16S rRNA similarity are poorly correlated ^31^. This suggests that when HGT does occur, the phylogenetic markers used to study prokaryotes will poorly represent microbial metabolism and thus the real (dis)similarity among microbiomes.

Despite all of these challenges, beta-diversity measures are regularly used to identify the factors that shape microbiota. The earliest attempts to understand microbiome assembly used gel electrophoresis–based community fingerprinting methods, such as terminal-restriction fragment length polymorphism (T-RFLP) analysis or (automated) ribosomal intergenic spacer analysis (RISA and ARISA). Using RISA and T-RFLP, for example, Micallef and colleagues ^6^ found that inbred accessions of *Arabidopsis thaliana* host distinct bacterial communities in the rhizosphere. In similar analyses, Bodenhausen and colleagues ^8^ and other authors ^32^ have shown that the leaf microbiome of *A. thaliana* also differs among inbred lines and that mutants defective in the leaf cuticle host altered microbial communities. Over the last decade, as high-throughput sequencing approaches have become more common, these gel-based approaches have largely been replaced by the sequencing of multiplexed amplicon libraries.

Laboratory studies have demonstrated that host genes influence microbial assembly ^6– 8, 17^, which raises the question of whether host effects can also be detected in natural settings, where organisms simultaneously confront abiotic and biotic stresses. Indeed, Redford and colleagues ^33^ found that trees of the same species have more similar leaf bacteriomes than trees of different species. Consistent with the results of Micallef and colleagues ^6^ and others ^8, 9^, inbred accessions of *A. thaliana* have likewise been shown to host distinct microbial communities in field settings. Specifically, both bacteria and fungi in the leaf ^34^ and root ^18^ microbiome of *Arabidopsis* are influenced by host genes. Moreover, it is now clear that genome-wide association studies (GWASs) can be used to shed light on the processes shaping microbiome assembly. For example, several promising candidate genes have been identified by using the sample coordinates from principal components analysis as phenotypes in GWASs. The top single-nucleotide polymorphisms (SNPs) associated with leaf fungi (PC1), for example, fall within *GLUCAN SYNTHASE LIKE 11* ( *GSL11*), a homolog of a gene ( *GSL5*) that deposits callose in the cell wall in response to fungal infection ^35^. Similarly, fungi in the roots (PC1) of *A. thaliana* are associated with promising candidate loci, including *PECTIN METHYLESTERASE 26* ( *PME26*) and its neighbor *PME3* ^18^. Like cellulose, pectin and callose are polysaccharides that play critical roles in cell-wall integrity.

Much of what we know about animal microbial communities comes from diverse human microbiome projects. For example, Blekhman and colleagues ^36^ used data from the Human Microbiome Project to explore microbiome habitats distributed across the body. This revealed that host genome similarity, which was cleverly estimated by using host contaminant reads, is moderately correlated ( *R* ^2^ ~ 0.19) with beta diversity in the stool microbiome. Unfortunately, the small sample size of this study (n = 93 individuals) and the failure to correct for population structure have made it difficult to interpret the results from their GWAS. However, the gut microbiomes of much larger human diversity panels are now being characterized. As an example, Wang and colleagues ^11^ used a classic epidemiological approach to investigate the gut microbiomes of a large northern European cohort (n = 1812). In that study, the authors were able to attribute approximately 10% of the variation in the microbiota to genetic differences among individuals. Differences in age, gender, body mass index, and smoking status explained about 9% of the overall variation, and controlling for these covariates in GWASs helped fine-map vitamin D receptor ( *VDR*) among the top candidate genes. Follow-up research with a mouse model (Vdr ^−/−^) has confirmed the role of *VDR* and helped in understanding its role in host-microbiome cross-talk through bile-acid sensing. Wang and colleagues have established the Microbiome Genome consortium ^37^ to further investigate the role of human genetics in shaping gut microbiota and the role of the microbiome in human disease. This project now has data for over 19,000 subjects for which rich metadata (for example, smoking status, age, and weight) are also being generated. The size of this cohort, and the accompanying resources, should further aid in understanding the genetic architecture of the microbiome.

Analyses of beta diversity for both plant and animal microbiomes have greatly improved our understanding of the factors that shape microbiota. Despite clear progress, however, the emerging view from this research is that environmental factors are more influential than host genes in shaping microbiomes (for example, as discussed in 16). In particular, it is difficult to detect host effects when environmental factors are not explicitly controlled for or taken into account during analysis ^11, 38, 39^. It is arguable that dispersal limitation and HGT should also be considered when designing experiments. Indeed, one should question the biological relevance of many microbiome traits (to begin with) before sequencing marker genes (for example, 16S rRNA) that poorly represent the phenotypic diversity of communities shaped by HGT ^31^. In the following sections, we discuss whether, despite these concerns, other microbiome traits are heritable.

### Alpha diversity

Alpha diversity describes local diversity, or the diversity within a sample (or habitat) ^40^. As is the case with beta diversity, there are multiple ways to characterize alpha diversity.

Perhaps the most widely known measure of alpha diversity is Shannon’s diversity index ^41^, which estimates the uncertainty in a sampling process by weighing the informativeness of elements in a series (for community and microbial ecologists, the elements are species) on the basis of their observed frequencies. This entropy is then exponentiated to estimate the effective number of species within a community, or true alpha diversity ^42^. The widespread use of Shannon’s index in ecology stems from the fact that it is biased toward neither common nor rare species. Despite this intuitive appeal, however, the phylogenetic markers that are used in most studies—sections of bacterial 16S rRNA ^43^ and the ITS regions of fungi ^44^—vary widely in gene copy number across species. This means that Shannon’s index is inappropriate unless corrective steps are taken ^24^ (for example, dividing each species’ abundance by its predicted number of marker gene copies) that are difficult to apply to many taxa.

Many researchers instead focus on species richness, the number of taxa within a sample, to understand alpha diversity. Species richness does not take abundance into account, and it can be investigated without rarefaction by using long-established statistical models (for example, negative binomial or quasi-Poisson generalized linear models) that allow one to adjust for differences in the number of sequences among samples by using “offsets” to model the exposure rate. This simplicity, combined with its low statistical burden, has made richness a popular phenotype.

As an example, bacterial richness in the maize rhizosphere ^38^ is shaped by genetic differences among accessions ( *R* ^2^ = 19%), as are the number of bacterial (heritability [ *H* ^2^] = 0.57) and fungal ( *H* ^2^ = 0.47) taxa in the root microbiome of *A. thaliana* ^18^. Of course, bacteria and fungi co-occur in many microbiomes, which suggests that they should be combined in analyses when appropriate. In the case of the root microbiome of *A. thaliana*, combining bacterial and fungal richness data enabled Bergelson and colleagues ^18^ to fine-map *CELLULASE1* and other promising candidate genes implicated in immunity. The top candidate SNPs fall nearby the disease resistance gene *NPR1* ^45^, which was not mapped in either of the marginal analyses.

Another measure of alpha diversity is Faith’s phylogenetic diversity (PD) ^46^, which uses phylogenetic trees generated with molecular or cladistic data to estimate diversity. Like species richness, Faith’s PD does not take “abundance” into account and thus avoids the marker gene copy-number problem. Faith’s PD has become a popular measure in conservation biology, where geographic regions with higher phylogenetic diversity are considered to be more diverse (and thus more valuable) than communities of closely related species. In the case of microbiome studies, analyses of Faith’s PD have revealed that the gut bacterial communities of both wild mouse ^47^ and humans ^48^ are heritable (for example, human *H* ^2^ = 0.37). Curiously, GWASs of the wild mouse ^47^ and human gut microbiome ^11^ have pinpointed the same candidate gene: *CSMD1*.

### Individual taxa

Characterizing microbiome beta and alpha diversity has greatly improved our understanding of the overall processes that shape microbial communities. Although microbes interact in these communities, many researchers are nevertheless interested in the presence/absence and abundances of individual taxa and whether individual taxa are also shaped by host genes.

As an example in plants, Walters and colleagues ^13^ recently identified 143 heritable operational taxonomic units (OTUs) in the rhizosphere of maize. Overall, the broad-sense *H* ^2^ of these taxa were low (15 < *H* ^2^ < 25%) compared with taxa in the phyllosphere of maize ^49^, which raises the question of whether these two habitats are differentially affected by environmental effects and host genes. Support for this hypothesis comes from work in *Boechera* ^50^, which similarly suggests that leaf microbiomes are more affected by host genes than root microbiomes.

In the case of the human gut microbiome, Goodrich and colleagues ^3, 12^ have identified several heritable bacterial groups. A member of the most highly heritable ( *H* ^2^ = 0.42) taxon, the Christensenellaceae (specifically, *Christensenella minuta*), has been shown to reduce weight gain in transplantation experiments with germfree recipient mice ^3^. Moreover, network analyses have revealed that the Christensenellaceae are the key group (based on node “degree”, or the number of network connections) in a sub-network of several heritable bacterial groups within the gut microbiome. This suggests that host genes may indirectly shape microbial communities through more direct interactions with key taxa (that is, “hub points”) in microbial community networks.

Genotype-by-environment interactions (GxEs) are widely believed to shape host microbiome traits, but the prevalence of GxEs remains unclear. The best-characterized example involves the lactase enzyme, which is encoded by the *LCT* locus. Polymorphisms at the human *LCT* locus determine the ability of adult humans to digest milk. These variants, however, not only are linked to lactase persistence but also with the abundance of the genus *Bifidobacterium* in the human gut microbiome. Strains of *Bifidobacterium* that can metabolize lactose are found in (significantly) increasing abundance with the increasing consumption of dairy products in the microbiomes of individuals homozygous for the haplotype associated with hypolactasia (lactose intolerance) in Europeans; this is strong evidence of a GxE ^10^.

Most microbial communities contain a large number of taxa, and the number increases with increasing sample size as rare taxa are discovered. Although this poses challenges for beta diversity (see above), studies of individual taxa often focus on the most heavily sequenced taxa (for example, the top 100 species) to improve speed and reduce the burden of multiple testing. So it is perhaps surprising that recent studies of bacteria have sought to examine both the heritability of individual taxa and (separately) the same data ** binned at increasingly higher taxonomic levels. As discussed above, prokaryotes experience high rates of HGT and gene loss, which suggests that their physiology is not conserved enough to justify the increased (statistical) burden of testing taxa at both low (for example, genus) and high (for example, phylum) taxonomic levels.

### Microbial metabolism 

As described above, amplicon-based analyses of beta diversity, alpha diversity, and the abundances of individual taxa are hampered in varying degrees by both HGT and biases introduced during PCR and sequencing ^25, 26^. One solution is to directly sequence microbial genes or RNA by using metagenome shotgun sequencing or transcriptomics. The key advantage of these two approaches is that the metabolic potential of the microbiome is directly measured rather than predicted from marker gene sequences. For studies focused on understanding community assembly processes, these untargeted sequencing approaches also have the potential to characterize species membership in a less biased and PCR-free manner while enabling the identification of traditionally overlooked groups (oomycetes, viruses, Cyanobacteria, and so on).

Among the disadvantages of metagenomes and metatranscriptomes is that they are expensive to generate, which is perhaps why they are rarely characterized outside of human and crop research. Moreover, host sequences may be enriched in some habitats (for example, 51, 52), resulting in contamination that further increase the costs of sequencing. The studies that have been performed so far have shown that microbial metabolism varies widely among species grown in the same environment, suggesting that hosts do affect microbial gene expression in some manner. As an example, enzymes involved in nitrate reduction are more highly expressed in the wheat than cucumber microbiome of plants grown in a randomized experiment ^53^. In the cucumber microbiome, enzymes associated with sulfur assimilation and pectin degradation are upregulated relative to wheat. As noted above, pectin, which is generally more abundant in the cell walls of dicots than monocots ^54^, is one of the polysaccharides that regulate host cell-wall integrity.

It is unclear how heritable individual microbial enzymatic categories and pathways are, but the limited number of studies that have explored the relationship between microbial metabolism and host variation have identified promising associations, although the abundances of taxa and microbiome functions appear to be influenced by different host genes ^10^. This is consistent with an emerging theme from microbial research: the microbial composition of samples is a poor proxy of microbial gene expression within the same samples and this is due in part to HGT ^55^. For example, the inner leaves of bromeliads form water traps that in turn form aquatic ecosystems. By sequencing the bacteria and the microbial genes within these aquatic islands, Louca and colleagues demonstrated that, despite hosting highly diverse bacterial communities, the genes expressed within these communities are remarkably similar ^56^. If a microbial community experiences high rates of HGT, it will be far more informative to characterize microbial metabolism than to sequence marker genes that are, in effect, decoupled from the genomes that they are intended to represent.

## Future research perspectives

Research in the past decade has illustrated a close relationship between a host’s microbiome and health ^3, 5^, which naturally leads to questions about how these communities form and whether host genes play a role. In this review, we summarize recent research focused on this topic while discussing whether the choice of microbiome traits affects our understanding of these communities.

What is the most meaningful way to summarize the microbiome? The most common approach is to characterize the overall similarity of microbiomes by using beta-diversity measures. These studies often find that microbiomes vary dramatically among conspecifics that live in different areas ^13, 50, 57^, which leads to the reasonable conclusion that spatially varying environmental factors differentially shape these communities. However, in most studies, it is unclear whether environmental factors are confounded with ecological processes, such as ecological drift and dispersal limitation. Whatever the research focus, if everything is *not* everywhere, approaches based on beta-diversity will be underpowered when samples from distant geographic regions are under consideration.

In comparison, analyses of individual taxa ^13, 48, 49^ and species richness ^18, 38^ have detected (modestly more) heritable components of the microbiome ^18, 49^. Despite these promising results, however, it should be noted that analyses of alpha diversity and individual OTUs share many of the same problems as beta diversity, including whether microbiomes distributed across the environment experience the same exposure rate (that is, due to dispersal limitation). Confounding factors are regularly included as covariates to study human diseases, and the strategies used in epidemiology ^58^ probably deserve more attention in the study of host microbiota ^11, 59^.

A major limitation of current host microbiome research is the fact that microbes are often shaped by HGT but characterized with marker genes (for example, 16S rRNA) that fail to distinguish among genetically divergent strains ^60^. As we move forward in this research, it will be critical to determine whether metagenomic or metatranscriptomic studies, which are unaffected by HGT, can uncover additional components of heritability.
